# Jejunal Ulcer Caused by* Schistosoma japonicum*

**DOI:** 10.1155/2019/8356438

**Published:** 2019-03-31

**Authors:** Daniela Jaqueline Rivadeneira, Hesheng Luo

**Affiliations:** Department of Gastroenterology, Renmin Hospital of Wuhan University, Wuhan 430060, China

## Abstract

Intestinal schistosomiasis can be caused by the trematodes* Schistosoma japonicum* that mainly exists in East Asia or the* S. mansoni *in Africa and South America. The adult worms of* S. japonicum* live in the mesenteric veins and excrete eggs that circulate to the liver and colon; the eggs migrate through the intestinal wall and pass out with the stool. Here, we report a case of jejunal ulcer caused by the infection of* Schistosoma japonicum*. A 63-year-old woman from Wuhan, China, was admitted with left quadrant abdominal pain and weight loss for more than 6 months. The patient's computerized tomography reported cirrhotic liver changes, jejunal wall edema, and narrowed lumen; the upper enteroscopy corroborated these findings with the presence of several jejunal ulcers and edema. The pathology report showed chronic inflammation with ulcerative changes and* S. japonicum* eggs deposition. Schistosomiasis is one of the neglected tropical diseases that affect the poorest. Although a great improvement has been made to control it, there is a lot of work that remains to be fulfilled.

## 1. Introduction

Neglected tropical diseases (NTD) are caused by infectious and parasitic agents that primarily affect the poorest and most vulnerable populations in the world [1]. Currently, the NTD are affecting 41.4 million people, causing approximately 35,000 deaths per day worldwide [2]. By 2014, the World Health Organization (WHO) reported that 258 million people worldwide were infected with trematodes of the genus* Schistosoma* and required treatment and preventive regime [3]. These “neglected” diseases are due to underfinancing and low recognition by the pharmaceutical industries [4, 5].

Schistosomiasis is considered the third most devastating tropical disease in Africa, South America, the Caribbean, Middle East, and Asia [6, 7]. The 2015 death estimates due to schistosomiasis need to be reassessed, as they vary between 24,067 and 200,000 globally per year [8].* S. japonicum *and* S. mekongi* are found in the Eastern Hemisphere [9]. In China,* S. japonicum* is found along the Yangtze River Basin, and according to a Chinese report for the WHO,* S. japonicum* infects 45 species of animals and remains endemic in seven provinces [10]. China is among the target countries to eliminate schistosomiasis according to the Regional Action Plan for Neglected Tropical Diseases in the Western Pacific (2012-2016), and according to the Ministry of Health, by the end of 2015, China aims to reach the criteria of transmission control threshold of <1% in the lake and marshland provinces and reach transmission interruption threshold in hilly provinces of Sichuan and Yunnan [10, 11].

The physiopathology of chronic schistosomiasis results from the eggs lodging, inducing immune response, granuloma formation, fibrotic changes, and damaging the organs and tissues of the human host.* S. japonicum* and* S. mansoni* adult worms reside in the mesenteric veins and excrete eggs that primarily circulate to the liver and intestines; the eggs migrate through the intestinal submucosa and mucosa, turn into the lumen, and pass out with the feces [12]. In the liver, the chronic progression causes periportal fibrosis, and it will lead to portal hypertension with all the cirrhosis complications if left untreated [12, 13]. Chronic schistosomiasis could be present from months to years after primary exposure; some do not have a clear history of acute phase or just mild and unspecific symptoms.* S. japonicum *or* S. mansoni* causes intestinal manifestations with fatigue, abdominal pain, cramping, anorexia, diarrhea, and dysentery [14]. If the infestation is heavy, the symptoms will be severe and may lead to intestinal ulceration, bleeding, anemia, intestinal polyps, dysplasia, and even bowel strictures [15].

The methods that are being used for schistosomiasis diagnosis depend on the target population and the* Schistosoma* species, but among people living in endemic areas, the approach should be through egg and antigen detection [16]. Another test is the real-time PCR; in stool samples, it can be 94% sensitive and 99.9% specific and may offer added value in diagnosing imported schistosomiasis [17]. The genus-specific PCR methods can detect and perform very well with feces and urine, but not with serum [18].

The treatment for schistosomiasis has important objectives such as reversing acute or early chronic disease and preventing complications. The drug of choice has always been praziquantel with a cure rate up to 90% after one regimen [19].

Prevention strategies to control schistosomiasis in endemic areas include water sanitation, minimizing contact with fresh water, community health-education, eradication of snail species, domestic animals care and treatment, proper work equipment to people at risk of exposition, and mass treatment [10, 19]. We present a case of chronic schistosomiasis with an unusual manifestation of* S. japonicum *in the small intestine.

## 2. Case Report

A 63-year-old Chinese woman from Wuhan was admitted to the First Affiliated Hospital of Wuhan University in October 2017 with left quadrant abdominal pain and weight loss for more than 6 months. The pain exacerbated after eating and was accompanied with abdominal distension, belching, and reduced flatus. The patient referred was taking traditional Chinese medicine that temporarily relieved the symptoms. She had previous history of hypertension with a poor control. She underwent colonoscopy examination in a local hospital and verbally reported no pathologic findings. The positive findings at the physical examination were the mesogastric tenderness and an enlarged spleen.

**Figure 1:**
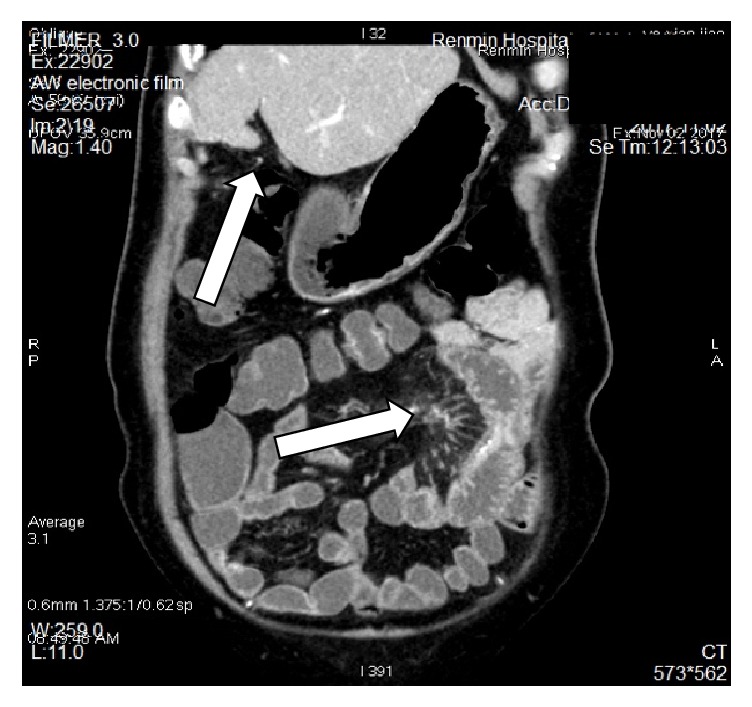
CT scan showing atrophy of the right liver lobe with hypertrophy of the left and caudate lobes, mesenteric edema.

**Figure 2:**
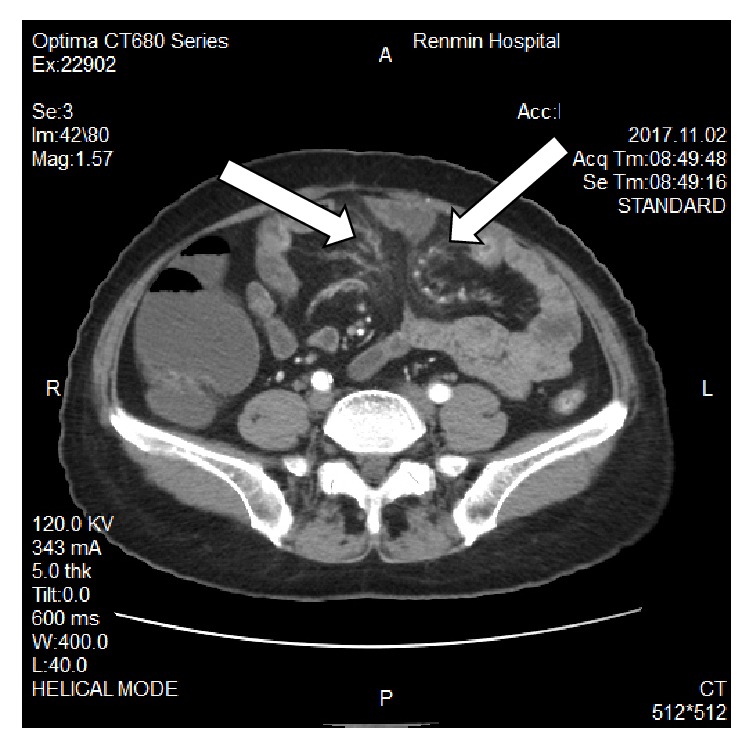
CT scan showing mesenteric edema, mesenteric fat blurred.

**Figure 3:**
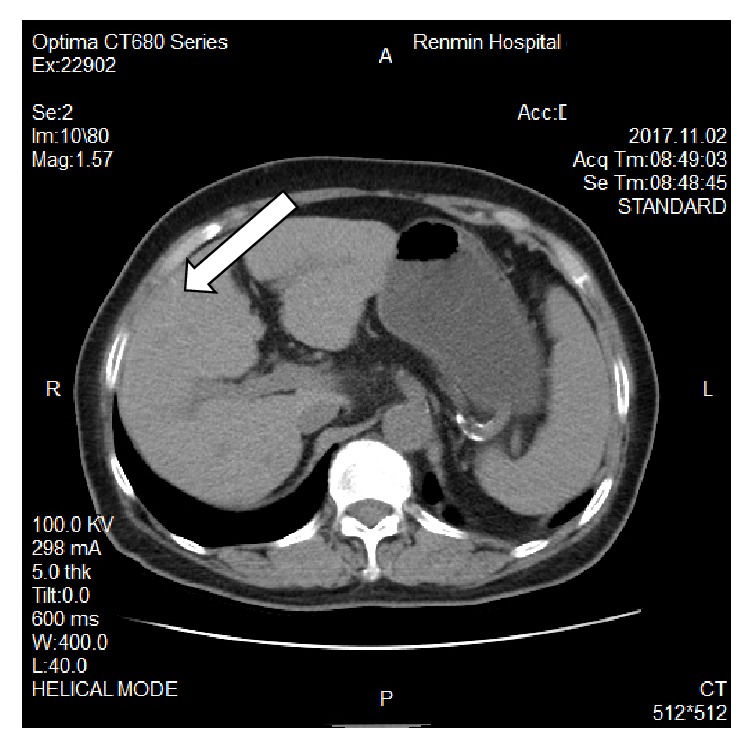
CT scan showing a linear calcification consistent with liver fibrosis due to* S. japonicum*.

**Figure 4:**
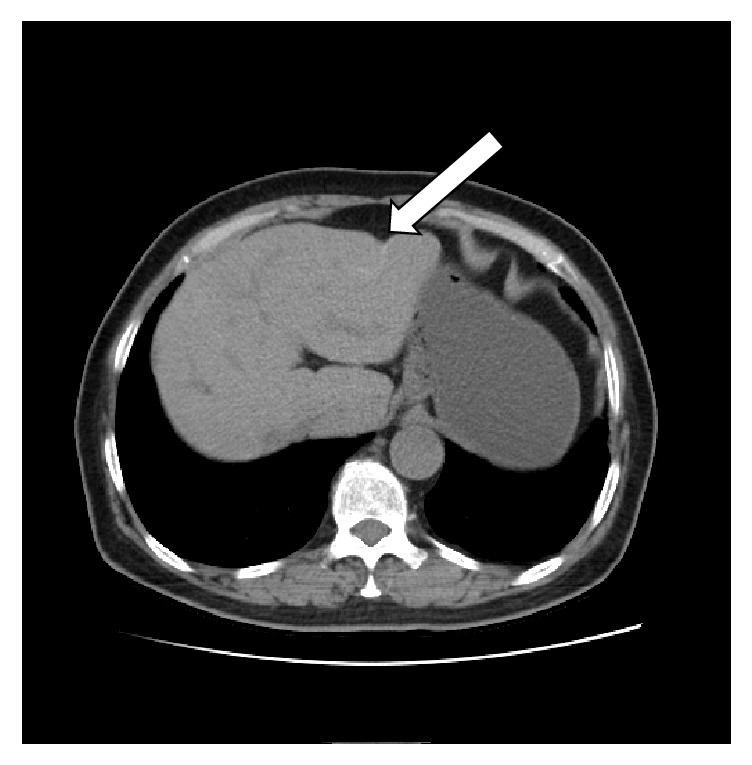
CT scan showing a linear calcification consistent with liver fibrosis due to* S. japonicum*.

**Figure 5:**
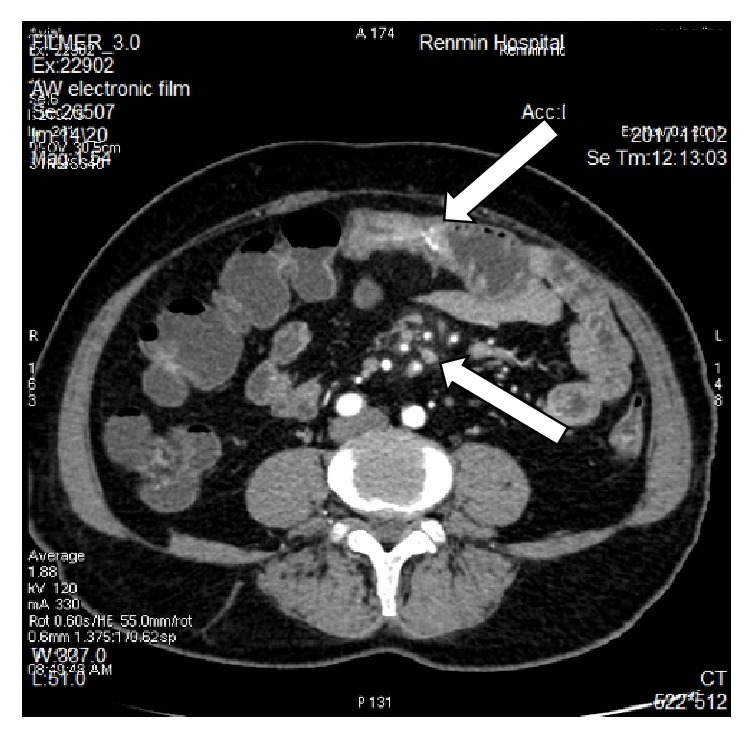
CT scan showing thickened intestinal wall, enlarged lymph nodes.

**Figure 6:**
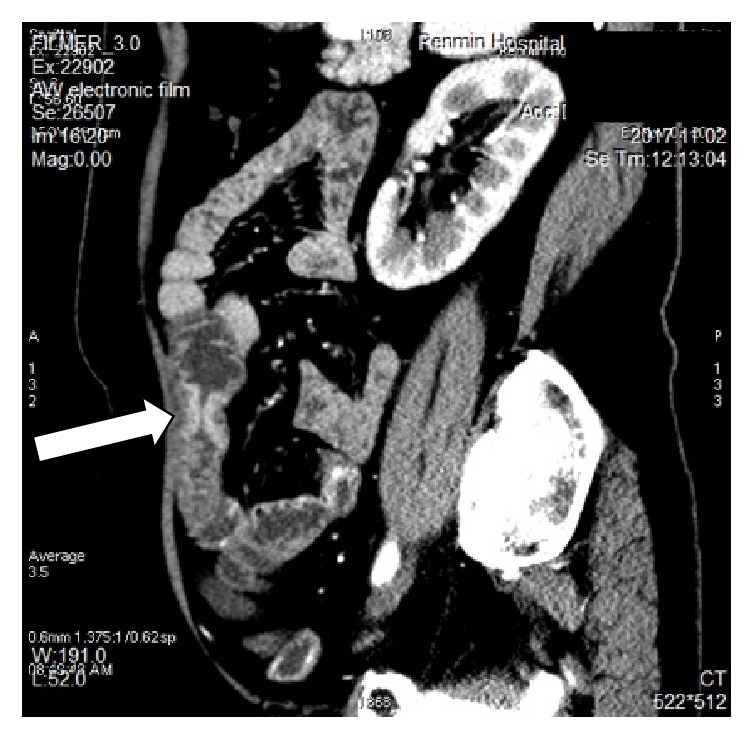
CT scan showing thickened intestinal wall and calcification.

The blood laboratory examination reported only mild hypokalemia; the urinary and stool reports were normal. The enhanced computerized tomography showed splenomegaly, the jejunal wall with edema and a narrowed lumen, enlarged lymph nodes, mesenteric edema; the liver cleft was widened with atrophy of the right liver lobe and hypertrophy of the left and caudate lobes; calcification and periportal fibrosis signs were compatible with hepatic schistosomiasis (Figures 1–6).

**Figure 7:**
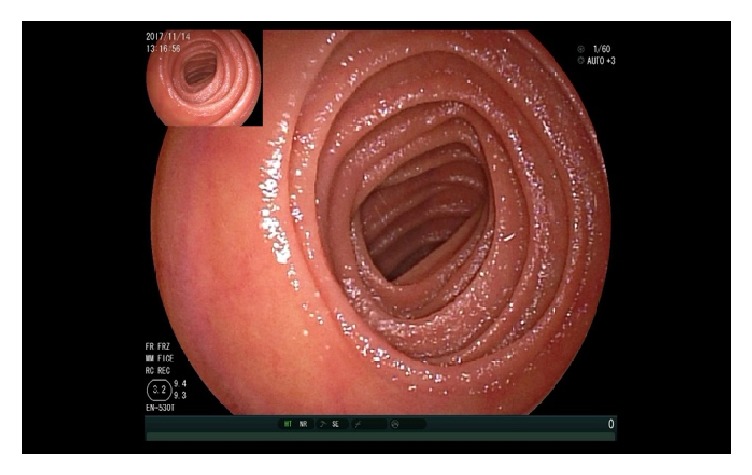
Enteroscopy showing normal jejunal mucosa.

**Figure 8:**
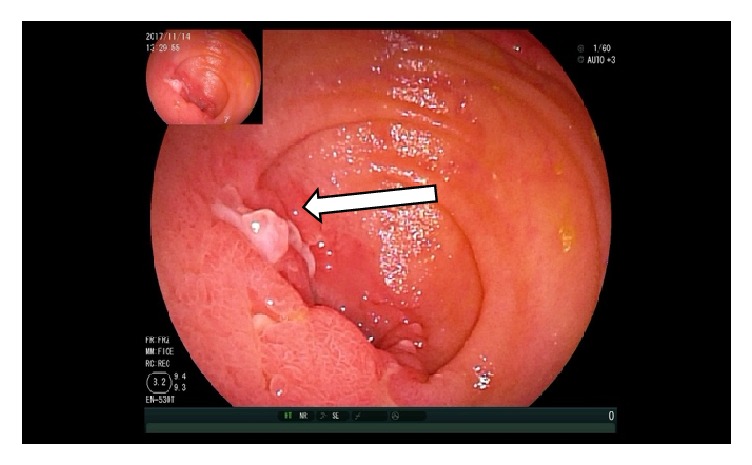
Enteroscopy showing a 3.0 x 2.0 cm size jejunal ulcer.

**Figure 9:**
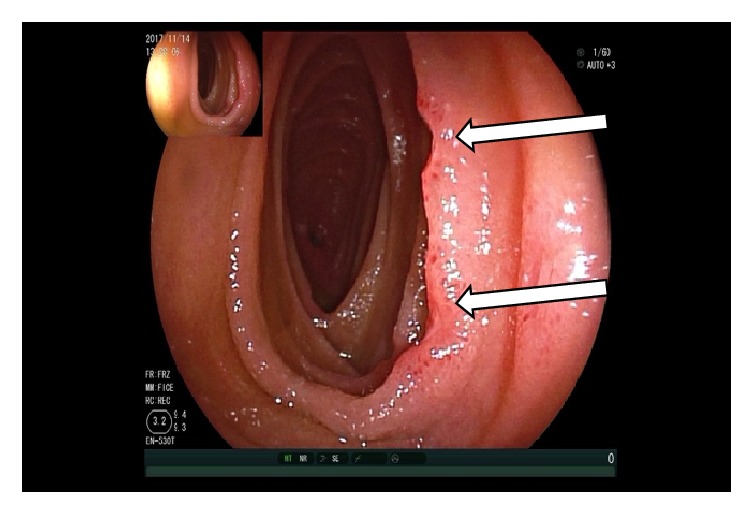
Enteroscopy showing several jejunal ulcers.

**Figure 10:**
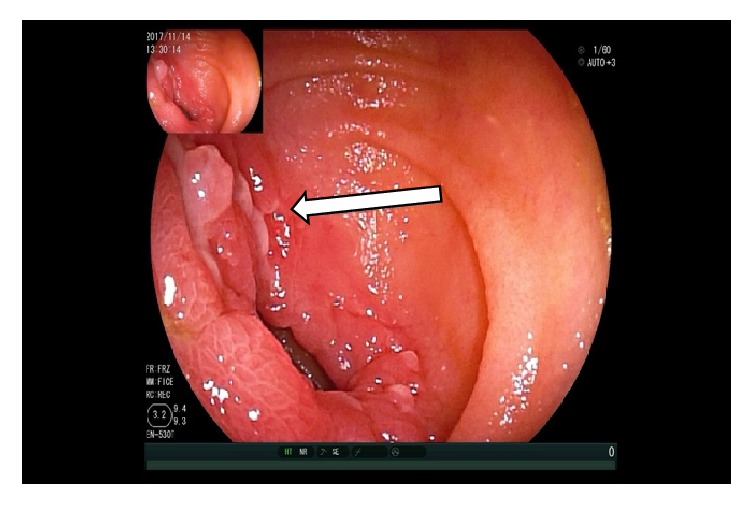
Enteroscopy showing a close view of the 3.0 x 2.0 cm size jejunal ulcer.

At the moment of the double balloon enteroscopy, the patient's blood pressure was not stable and we could not reexamine the colon. The upper enteroscopy showed at 150 cm distal to the Treitz ligament a 3.0 x 2.0 cm size ulcer with bottom white coating, peripheral mucosal hyperemia, and edema occupying 2/3 of the lumen so that the endoscope could not pass through. Four biopsies were taken from the ulcer (Figures 7–10). And 20 cm proximal from the ulcer, there were many scattered irregular ulcers from where two biopsies were taken.

**Figure 11:**
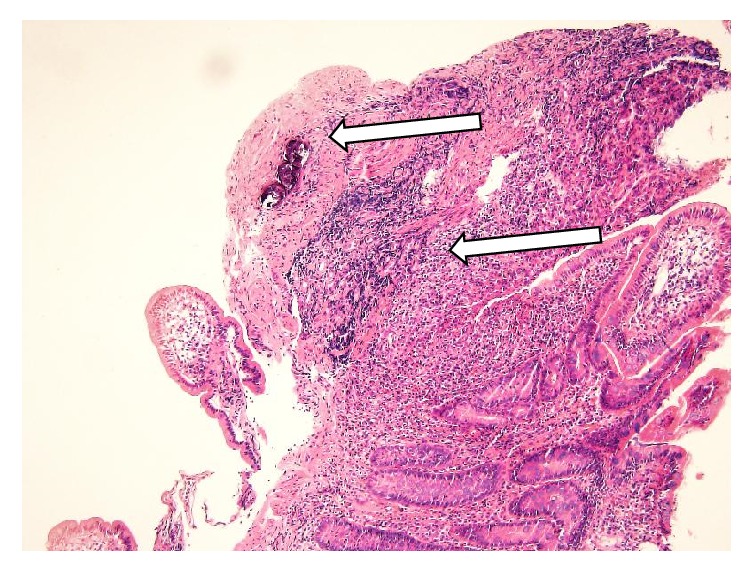
100x histopathology of jejunal mucosa showing chronic inflammation, ulcerative changes,* S. japonicum *eggs deposition.

**Figure 12:**
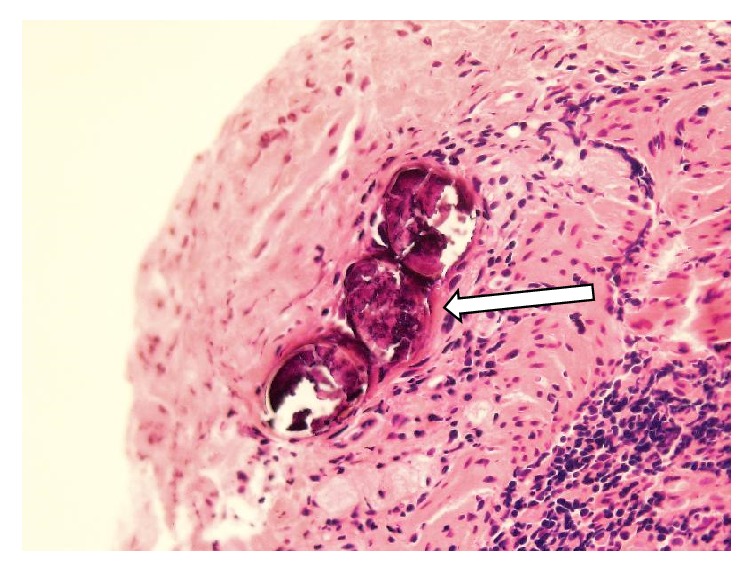
400x histopathology showing old* S. japonicum *eggs deposition.

The histopathological examination reported chronic jejunal inflammation with ulcerative changes and old* S. japonicum* eggs deposition (Figures 11 and 12).

During hospitalization, the patient received symptomatic treatment with intravenous fluids, electrolytes, antispasmodic medication, and proton-pump inhibitors. Unfortunately, the patient requested for a voluntary discharge from the hospital and decided to continue her treatment and follow-up at a local hospital.

## 3. Discussion

The importance of this case is the rare finding of jejunal ulcers due to* S. japonicum *infection, causing intestinal symptoms like abdominal pain and weight loss. The patient's CT scan corroborated chronic liver changes due to schistosomiasis; the upper enteroscopy and histopathological exam reported the unusual affection of the jejunal mucosa and diagnosed chronic infection with* S. japonicum*. The patient had abdominal pain for six months; she went to another hospital before arriving to ours and she did not get a correct diagnosis. This case teaches us that chronic abdominal pain has a broad range of differential diagnosis and that a well-done anamnesis is very important. In endemic areas like China where schistosomiasis remains prevalent, it should be within the differential diagnosis, and when a patient like ours shows abdominal symptoms, intestinal schistosomiasis should not be forgotten. When medical physicians are considering intestinal schistosomiasis, they should look for small and large intestine infection, and when endoscopists find intestinal ulcers, they should keep in mind this neglected tropical disease.

Despite the governmental and WHO efforts, more attention and support should be paid to this endemic disease, ensuring that the affected population not only receives suitable treatment, but also the education and preventive measures that will not expose them to suffer from this parasitic disease. More support should be given to control the transmission, prevent resistance to praziquantel, and develop a vaccine accessible to people at risk of schistosomiasis.

We encourage the education to endemic areas' population to seek medical advice when gastrointestinal manifestations occur, to medical physicians to consider intestinal schistosomiasis as a differential diagnosis when encountering gastrointestinal symptoms, to keep in mind that* S. japonicum* can also affect the small intestine and that if there are indications for enteroscopy examination, the patients should do it. Small intestine schistosomiasis is a rare manifestation of this tropical disease, but if there exist epidemiological risk, gastrointestinal affection, and ulcers, then small intestinal schistosomiasis should be considered as a possible diagnosis.
